# Primary Versus Salvage Distal Femoral Endoprosthetic Replacement Following Native Distal Femur Fracture: A Systematic Review and Meta-Analysis

**DOI:** 10.1016/j.artd.2025.101939

**Published:** 2026-01-07

**Authors:** Lucas Ho, Navnit S. Makaram, Catherine James, Chryssa Neo, Nick D. Clement, Chloe E.H. Scott

**Affiliations:** a Edinburgh Medical School, The University of Edinburgh, Edinburgh, UK; b Edinburgh Orthopaedics, Royal Infirmary of Edinburgh, Edinburgh, UK

**Keywords:** Distal femoral fracture, Distal femoral endoprosthetic replacement, Primary, Salvage, Arthroplasty, Native

## Abstract

**Background:**

Surgical management of native distal femoral fractures (DFFs) in elderly patients includes open reduction and internal fixation (ORIF) or distal femoral endoprosthetic replacement (DFR). When ORIF is complicated by nonunion or fixation failure, salvage DFR (sDFR) may be required. The comparative outcomes of primary DFR (pDFR) vs sDFR remain unclear. This systematic review and meta-analysis aimed to assess the quality of published literature and compared clinical and functional outcomes between pDFR and sDFR for native DFFs.

**Methods:**

MEDLINE, Embase, and Cochrane databases were searched from inception to April 2024. Studies investigating outcomes of pDFR or sDFR following native DFFs were included. Studies evaluating periprosthetic fractures, oncologic indications, or primary arthritis were excluded. Twelve studies comprising 281 patients (230 pDFR, 51 sDFR) were included.

**Results:**

Patients undergoing pDFR were significantly older (mean 79.3 vs 64.9 years; *P* < .001) and more comorbid (American Society of Anesthesiologists score: mean 2.99 vs 2.34; *P* < .001). Despite this, pDFR was associated with significantly lower reoperation (12.2% vs 23.5%; *P* = .04) and complication rates (15.7% vs 43.1%; *P* < .001) compared to sDFR. 1-year mortality rate was higher in the pDFR cohort (10.4% vs 2.0%). Functional outcomes were marginally lower in pDFR, although this was not statistically significant (76.3 vs 80.7%; *P* = .09).

**Conclusions:**

sDFR following failed fixation of native DFFs was associated with nearly twice the risk of reoperation and postoperative complications compared to pDFR, despite being performed in a younger and less comorbid cohort. Elderly patients at risk of fixation failure may therefore benefit from pDFR.

## Introduction

Distal femoral fractures (DFFs) in older adults account for 3-6% of all femoral fractures [1], and their incidence is increasing, with an estimated incidence of 6.4 per 100,000 person-years [2]. DFFs present a significant clinical challenge, particularly in elderly patients, where comorbidities, poor bone stock, and limited rehabilitation potential contribute to higher complication rates and poor functional outcomes [3].

The management of DFFs has traditionally relied on open reduction and internal fixation (ORIF), using either locking plates and screws, retrograde intramedullary nails, or, more recently, a combination of these devices [4]. While ORIF preserves native bone, its effectiveness is limited in cases of severe comminution (articular or metaphyseal) and poor bone stock, and it carries a risk of fixation failure and nonunion of 20–24% in some studies [5,6]. These risks are further amplified in older patients where osteoporotic bone may struggle to provide sufficient support for internal fixation, compromising its ability to withstand axial and torsional loading and stabilize impacted articular segments. Furthermore, older patients may face difficulties adhering to restricted weight-bearing regimens due to baseline weakness and cognitive decline [7]. As with other periarticular fractures of weight-bearing joints in the elderly, such as tibial plateau fractures, both nonoperative management and ORIF carry a risk of later conversion to arthroplasty [8].

**Figure 1:**
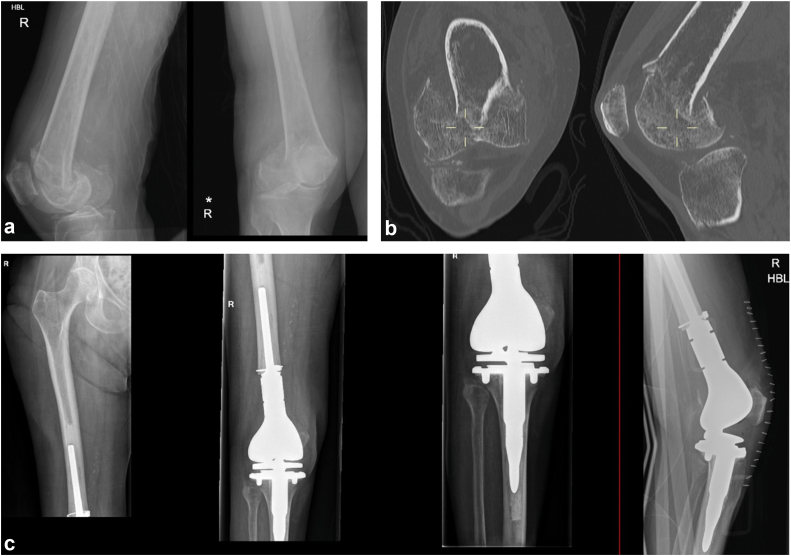
Anteroposterior and lateral radiographs of an AO 33C3 native distal femoral fracture in a 92-year-old female, managed with distal femoral endoprosthetic replacement. (a) Anteroposterior and lateral radiographs of distal femoral fracture. (b) Relevant coronal and sagittal computed tomography images of the same distal femoral fracture. (c) Anteroposterior and lateral postoperative radiographs following distal femoral endoprosthetic replacement in the same patient.

Distal femoral endoprosthetic replacement (DFR) has been advocated as a primary treatment option for selected elderly patients where the fracture sustained is unreconstructable due to severe comminution or poor bone stock (Fig. 1). Further proposed advantages include a lower reoperation rate (due to the elimination of risks of nonunion, post-traumatic arthritis, or fixation failure) and a shorter length of stay in hospital due to immediate unrestricted weight-bearing and potentially faster mobilization [8].

Despite the proposed advantages, DFR is associated with risks which have prevented its routine use for elderly DFFs, most notably deep infection, loosening and wear, limited further salvage options, and the financial cost of the implant [4]. Previous studies have primarily focused on comparing ORIF and DFR for periprosthetic DFFs or evaluating primary DFR (pDFR) and salvage DFR (sDFR) separately, and most studies are limited to small case series. Among the studies comparing ORIF and DFR for native DFFs, DFR has been associated with lower revision and reoperation rates compared to ORIF [6]. Where sDFR is performed following failed internal fixation, and surgery is often technically more complex due to scarring from previous surgeries and the approaches used, retained metalwork, poorer soft tissue conditions, and poorer patient general health. As a result, prior studies have reported high complication rates in the sDFR scenario [7,9]. However, the literature evaluating outcomes of pDFR compared with sDFR following initial fixation is limited, and it remains unclear whether pDFR in the setting of fragility-related fractures may be a superior option to sDFR following failed ORIF.

This systematic review and meta-analysis aimed to assess the quality of published evidence on primary vs salvage DFR for native DFF and to compare their pooled clinical and functional outcomes.

## Material and methods

This study was conducted in accordance with the Preferred Reporting Items for Systematic Reviews and Meta-Analyses guidelines [10] and was registered with PROSPERO (International Prospective Register of Systematic Reviews) (CRD42024537773). A comprehensive search of the MEDLINE, Embase, and Cochrane Central databases was performed to identify all studies assessing primary or salvage DFR following trauma, covering publications from inception to April 2024.

All abstracts identified in the initial search were independently assessed by 2 reviewers (L.H. and N.S.M.) to identify studies that met the inclusion criteria. Full-text reviews were conducted for potentially relevant articles, and any disagreements were resolved through consensus with the senior author (C.E.H.S.). Additional studies were identified through manual screening of reference lists from included articles. Data extraction from eligible studies was performed independently by 2 authors (L.H. and N.S.M.) and entered into R statistical software (R Core Team, Vienna, Austria). Extracted data included the number of patients and relevant patient demographics.

### Search terms

The search was performed on May 1, 2024, using the Medical Subject Headings (MeSH) terms: (“fem∗ endoprosthe∗” OR “fem∗ megaprosthe∗” OR “fem∗ revision∗” OR “endoprosthe∗ replacement∗” OR “fem∗ replacement∗” OR “fem∗ arthroplast∗”) AND “distal.”

### Eligibility criteria

Inclusion criteria comprised the following: prospective and retrospective studies, randomized and non-randomized comparative studies, and case series (>3 patients) investigating outcomes of pDFR for traumatic native DFFs or sDFR following any other primary treatment (nonoperative, ORIF, or external fixation) for traumatic DFFs (Table 1).

**Table 1:** Summary of inclusion and exclusion criteria.

Inclusion criteria	Exclusion criteria
Studies investigating outcomes of primary DFR as initial treatment for traumatic DFFs in native femurs	Studies investigating outcomes of DFR for conditions other than traumatic fractures (eg, pathologic fracture, malignancy, primary arthritis)
Studies investigating outcomes of salvage DFR following either nonoperative treatment or fixation as initial treatment for traumatic DFFs	Conference abstracts, book chapters, narrative and systematic reviews, technical notes, individual case reports
Studies published in the English language	Knee replacement revisions
Prospective or retrospective studies, case series (n > 3)	

n, number of patients.

Exclusion criteria comprised the following: studies not published in the English language; DFR performed for indications other than trauma (pathologic fracture, malignancy, primary arthritis); DFR performed for periprosthetic fracture; conference abstracts, book chapters, narrative or systematic reviews, technical notes, case reports, and case series with three or fewer patients; studies assessing proximal tibial fractures; studies assessing proximal or total femoral endoprosthetic replacement; studies assessing periprosthetic fractures; and studies comprising a heterogeneous cohort of DFFs where the data did not differentiate between acute traumatic fractures around the native joint and other indications for DFR (periprosthetic fracture, oncological) (Table 1).

### Literature search

**Figure 2:**
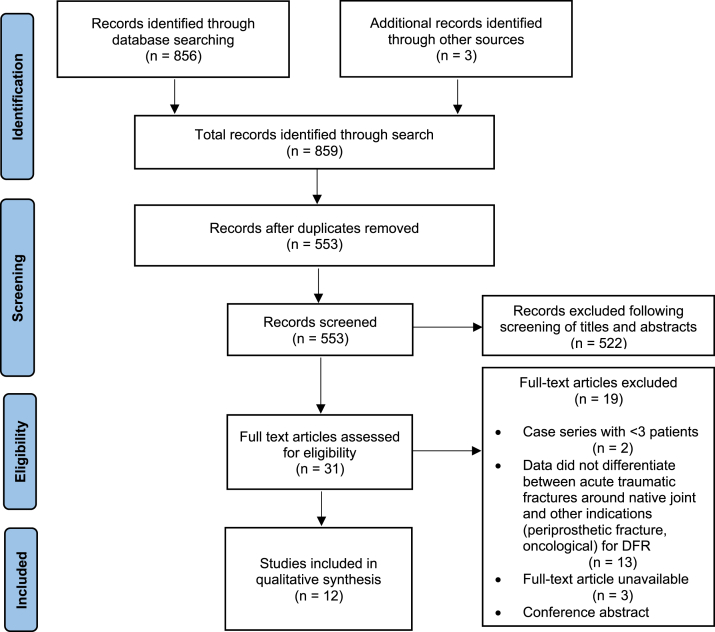
Preferred Reporting Items for Systematic Reviews and Meta-Analyses (PRISMA) flowchart for study inclusion.

The literature search identified 856 potentially relevant studies, of which 12 studies involving a total of 281 participants met the inclusion criteria and were included in the final qualitative analysis (Fig. 2). Of the 12 included studies, 10 were retrospective [[5], [6], [7], [8], [9],[11], [12], [13], [14], [15]] and 2 were randomized controlled trials (RCTs) [4,16]. There were 7 studies for pDFR [[4], [5], [6], [7], [8], [9],^16^] and 5 studies for sDFR [[11], [12], [13], [14], [15]]. Summary characteristics of the included studies are presented in Table 2.

**Table 2:** Summary of studies included assessing primary and salvage distal femoral endoprosthetic replacement (DFR) for traumatic distal femoral fractures.

Group	Author, year	Journal	Study design, LoE	N	% female	Mean age at DFR(y) (SD)	Mean LoS (d) (range)	Mean f/u (mo) (SD/Range)	ASA	Classn method	Classn. (n)	Mortality <1 y (n, %)	Revision/ Reoperation (n, %)
PrimaryDFR	Aebischer 2022	*Bone & Joint Journal*	Retrospective, III	153	84.8	76.1 (11.9)	NR	37.2 (0 to 216)	3.04	NR	NR	19 (12.5)	16 (10.0)
	Hart 2017	*Journal of Arthroplasty*	Retrospective, III	10	100.0	81.8 (5.2)	7.3 (4 to 12)	12.0^a^	NR	AO/OTA	33C (10)	1 (10.0)	1 (10.0)
	Hull 2019	*Bone & Joint Journal*	Randomized Controlled Trial, II	11	100.0	87.9 (4.9)	22.8 (SD 11.52)	5.5 (0.1 to 9)	NR	AO/OTA	33A (7) 33C (4)	1 (9.0)	1 (9.0)
	Bettin 2016	*Journal of Orthopaedic Trauma*	Retrospective, IV	18	66.7	77.1 (8.0)	11.0 (5 to 43)	2.2 (0.3 to 5.9)	3	AO/OTA	33B (4) 33C (14)	8 (44.4)^b^	4 (22.2)
	Gendya 2023	*Arthroplasty today*	Randomized Controlled Trial, II	16	80.0	69.0 (6.5)	NR	5.4 (0.1 to 12)	NR	AO/OTA	33A3/33C (16)	NR	4 (26.6)
	Tibbo 2022	*European Journal of Orthopaedic Surgery & Traumatology*	Retrospective, III	10	60.0	80.3 (10.4)	13.0 (9.8 to 17.5)	20.0 (2.2 to 48)	NR	AO/OTA	33C (10)	4 (40.0)^c^	1 (10.0)
	Caines 2023	*Journal of Orthopaedic Trauma*	Retrospective, IV	12	100.0	83.0 (9.0)	52.2	26.4	2.34	AO/OTA	33C (12)	0 (0)	1 (8.3)
	Overall primary DFR			230	84.2	79.3 (10.1)	14.4	31.4 (31.3)	2.99			21 (10.4)	28 (12.2)

	Author, year	Journal	Study design, LoE	n	% female	Mean age at DFR(y) (SD)	Mean LoS (d) (range)	Mean f/u (mo) (SD/Range)	NOS/ CRoB	Classn. Method	Classn. (n)	Mortality <1y (n, %)	Revision/ Reoperation (n, %)
SalvageDFR	Rajasekaran 2020	*International Orthopaedics*	Retrospective, IV	24	29.1	71.8 (4.2)	10.0 (8 to 17)	22.1 (10 to 43)	2.46	NR	NR	0 (0)	0 (0)
	Freedman 1995	*Journal of Orthopaedic Trauma*	Retrospective, III	3	100.0	65.0 (3.2)	16.0 (9 to 30)	32.6 (24 to 48)	NR	AO/OTA	33C1 (1)NR (2)	0 (0)	1 (33.3)
	Corap 2022	*BMC Musculoskeletal Disorders*	Retrospective, III	8	87.5	61.8 (18.5)	5.5 (SD 1.9)	46.8 (6.8 to 118.8)	2	NR	NR	0 (0)	7 (87.5)
	Vaishya 2011	*Knee Surgery, Sports Traumatology Arthroscopy*	Retrospective, IV	8	37.5	74.0 (4.2)	NR	48.0 (36 to 72)	NR	NR	NR	0 (0)	1 (12.5)
	Colatruglio 2023	*Archives of Trauma Research*	Retrospective, III	8	75.0	52.0 (6.1)	11.0 (6 to 32)	36.2 (SD 21.6)	NR	NR	NR	1 (12.5)	3 (37.5)
	Overall salvage DFR			51	50.9	64.9 (12.1)	12.6	41.4 (21.5)	2.34			1 (2.0)	12 (23.5)

LoE, level of evidence; n, number of patients; SD, standard deviation; LoS, length of stay; NR, not reported; f/u, follow-up; ASA, American Society of Anesthesiologists; NOS, Newcastle-Ottawa Scale; CRoB, cochrane risk of bias; Classn, classfication; AO/OTA, AO foundation/Orthopaedic Trauma Association.

a Only reported 1-year follow-up.

b Reported at an average of 4.7 years postop.

c Reported prior to 2-year follow-up time point; both studies were not included in the calculation of overall mortality < 1y.

### Outcome measures

The outcomes of interest included complication rates, functional outcome scores, length of stay, mortality, revision or reoperation rates, and etiology of revision or reoperation. Complications of interest comprised bone cement implantation syndrome, superficial and deep infection, wound breakdown, venous thromboembolism, dislocation, extensor mechanism disruption, aseptic loosening, periprosthetic fracture, stiffness requiring manipulation under anesthesia, critical care requirement, and any medical complications (respiratory, cardiac, neurological, or other).

### Data analyses

Summary statistics indicating the number of patients extracted from individual studies were obtained using counts, frequencies, and percentages. Weighted means were calculated for each outcome, and where available, standard deviations are stated. The relationship between groups of dichotomous variables was assessed using a chi-squared or Fisher’s exact tests. The relationship between continuous variables between groups was assessed using paired Student’s *t*-tests. All analyses were performed using Statistical Package for the Social Sciences (SPSS) 24 (IBM Corp, Armonk, NY) and R statistical software (R Core Team, Vienna, Austria). Heterogeneity was tested using the I^2^ index based on Cochran Q, with I^2^ >50% deemed heterogeneous. Means were represented with standard deviations and 95% confidence intervals (95% CI), weighted by sample size. Random-effects modeling was used to measure overall rates of intraoperative and postoperative complications and to assess functional outcomes in the primary and secondary DFR groups.

### Quality Assessment

The quality of included studies was assessed by 2 authors (L.H. and N.S.M.) using the Newcastle-Ottawa Scale [17] and Cochrane Risk of Bias 2 tool. [18]

**Figure S1:**
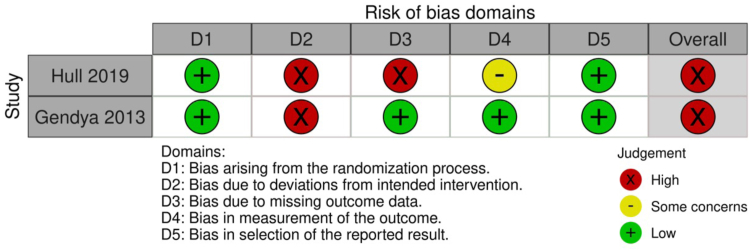
Quality assessment: Cochrane Risk of Bias.

The Newcastle-Ottawa Scale assesses study quality by evaluating the presence of each of the 9 items, assigning an overall quality score between 0 and 9. Categories include Selection (4 items), Comparability of cohorts (1 item), and Assessment of Outcome (3 items). Each item can be awarded a maximum of 1 star, except for Q5 where a maximum of 2 stars can be awarded. Studies are considered high quality when awarded 7 or more stars, moderate when awarded 4 to 6 stars, and poor when awarded 0 to 3 stars. There were 3 high-quality studies [5,6,9] and 7 moderate quality studies [7,8,[11], [12], [13], [14], [15]] (Table 3). Most studies did not use any control group for comparison. The Cochrane Risk of Bias 2 tool assesses the reliability of RCTs by evaluating six key domains: selection bias, performance bias, detection bias, attrition bias, reporting bias, and other bias. Each domain is rated as low, high, or unclear risk, and a global risk of bias assessment can be performed for each study. Both RCTs [4,16] were determined to have a high risk of bias, primarily due to the absence of blinding, which increases the potential for performance bias (Supplemental Figure 1).

**Table 3:** Quality assessment: Newcastle-Ottawa Scale.

Study, year	Selection				Comparability	Outcome			Score
	Representativeness of exposed cohort	Selection of nonexposed cohort	Ascertainment of exposure	Outcome not present at the start of the study		Assessment of outcomes	Length of follow-up	Adequacy of follow-up	
Aebischer 2022	a		a	a		a	a	a	6
Hart 2017	a	a	a	a	a	a	a		7
Bettin 2016	a		a	a		a	a	a	6
Tibbo 2022	a	a	a	a	a	a	a	a	8
Caines 2023	a	a	a	a	a	a	a	a	8
Rajasekeran 2020	a		a	a		a	a	a	6
Freedman 1995	a		a	a		a	a		5
Corap 2022	a		a	a		a	a		5
Vaishya 2011	a		a	a		a	a	a	6
Colatruglio 2023	a		a	a		a	a		5

a one score.

### Functional outcomes

Functional outcomes were assessed using the Oxford Knee Score and Knee Society Score in 7 studies, 6 of which were reported by studies in pDFR cohort (Table 4). All functional outcome data were reported at final follow-up. The Knee Society Score was the most commonly utilized functional outcome measure, appearing in 5 studies, while the Oxford Knee Score was used in 2 studies. To facilitate comparison and pooling of results, all scores were standardized as a percentage of their maximum possible value.

**Table 4:** Summary of functional outcomes by studies assessing primary and salvage distal femoral endoprosthetic replacement (DFR) for traumatic distal femoral fractures.

Primary DFR (n = 230)					Salvage DFR (n = 51)				
Study	n	Primary functional outcome score used	Mean value(SD/Range)	Score as percentage of best score possible [%] (SD/Range)	Study	n	Primary functional outcome score used	Mean value(SD/Range)	Score as percentage of best score possible [%] (SD/Range)
Aebischer et al. (2022)	153	NR	NR	NR	Rajasekaran et al. (2020)	24	KSS Knee Score	75.7 (63 to 88)	75.7 (63 to 88)
Hart et al. (2017)	10	NR	NR	NR	Freedman et al. (1995)	3	NR	NR	NR
Hull et al. (2019)	11	Oxford Knee Score	31.0 (30 to 32)	64.6 (63 to 67)	Corap et al. (2022)	8	NR	NR	NR
Bettin et al. (2016)	18	KSS Knee Score	85.7	85.7	Vaishya et al. (2011)	8	KSS Knee Score	86.0 (80 to 92)	86.0 (80 to 92)
Gendya et al. (2023)	16	KSS Knee Score	78.9 (23.2)	78.9 (23.2)	Colatruglio et al. (2023)	8	NR	NR	NR
Tibbo et al. (2022)	10	KSS Knee Score	80.0 (16)	80.0 (16)					
Caines et al. (2023)	12	Oxford Knee Score	34.8 (20 to 43)	72.5 (42 to 90)					
Overall score for primary DFR group				76.3	Overall score for salvage DFR group				80.7
Mean follow-up (mo)				31.4	Mean follow-up (mo)				41.4

n, number of patients; NR, not reported; SD, standard deviation.

### Implants

The type of implant used was reported in 6 of 7 studies assessing pDFR (219 of 230 cases) and in 4 of 5 studies assessing sDFR (43 of 51 cases). A total of 8 different implant types were used. The most commonly employed implants were the Global Modular Arthroplasty System (Stryker, Mahwah, NJ, USA), the Orthopaedic Salvage System (Zimmer Biomet, Warsaw, IN, USA), and the Modular Universal Tumour and Revision System (Implantcast GmBH, Buxtehude, Germany). The majority of implants were cemented (248 of 262 cases). Femoral stems were consistently cemented, most commonly using a retrograde, pressurized technique into the femoral canal. Tibial fixation, however, was more variable: some series used press-fit tibial stems with cement only under the tray, while others utilized a cemented long-stem tibial component.

## Results

### Cohort demographics

A total of 230 patients underwent pDFR for the primary management of DFF, and 51 patients underwent sDFR following initial fixation (internal or external) of DFF.

**Figure S2:**
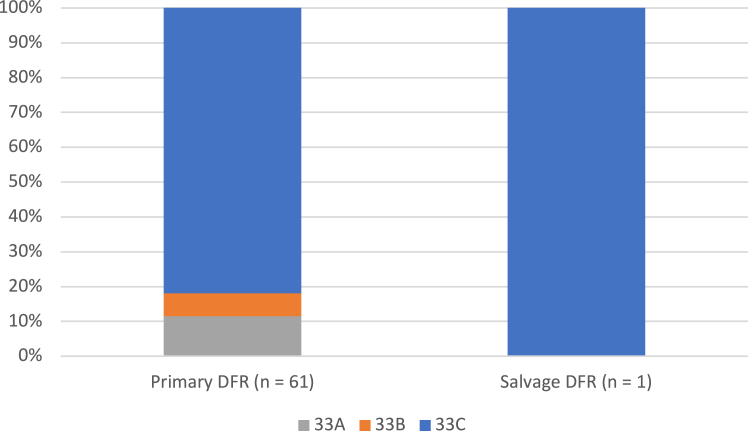
Bar chart showing fracture type (AO/OTA classification) in included studies. AO/OTA, AO foundation/Orthopaedic Trauma Association.

The pDFR cohort predominantly consisted of female patients, in contrast to the sDFR cohort, which had a more balanced gender distribution (84.2% vs 50.9%; *P* < .001; 95% CI: 20.4% to 49.4%). Patients in the pDFR cohort were significantly older at the time of DFR (mean 79.3 vs 64.9 years; *P* < .001; 95% CI: 10.8 to 17.9). The American Society of Anesthesiologists score was significantly higher in the pDFR cohort (mean 2.99 vs 2.34; *P* < .001; 95% CI: 0.5 to 0.8). The length of follow-up after DFR was significantly longer in sDFR cohort (31.4 vs 41.4 months; *P* < .05; 95% CI: 2.9 to 17.2). The length of stay was longer in the pDFR cohort, but this was not statistically significant (14.4 vs 12.6 days; *P* = .34; 95% CI: 0.5 to 3.1). Fracture type was reported using the AO foundation/Orthopaedic Trauma Association classification in 7 of the 12 included studies, 6 of which were reported by studies in pDFR cohort, the majority with type 33C fractures (83.3%) (Supplemental Figure 2).

### Mortality

Bettin et al. [7] reported mortality at an average of 4.7 years following pDFR. Tibbo et al. [6] reported mortality prior to 2 years of follow-up following pDFR. As such, both studies were excluded from the calculation of overall mortality within 1 year. Despite this exclusion, the pDFR cohort still exhibited a higher 1-year mortality rate, with 21 deaths (10.4%) compared to 1 death (2.0%) in the sDFR cohort.

### Revision/reoperation

The requirement for surgical reintervention (revision or reoperation) was higher in the sDFR cohort, with 28 reinterventions (12.2%) following pDFR compared to 12 reinterventions (23.5%) following sDFR (odds ratio: 0.45; 95% CI: 0.21 to 0.96; *P* = .04).

### Complications

Both intraoperative and postoperative complication rates were lower in the pDFR studies (Table 5). Intraoperative complications occurred in one (0.4%) pDFR case compared to 2 (3.9%) sDFR cases. The intraoperative complications included cardiac arrest and bone cement implantation syndrome. Postoperative complications were also significantly lower in the pDFR cohort, with 36 complications (15.7%) compared to 22 (43.1%) in the sDFR cohort (odds ratio: 0.24; 95% CI: 0.12 to 0.46; *P* < .001). Other postoperative complications included patellofemoral impingement in the setting of an unresurfaced patella requiring revision [6], stage 2 decubitus ulcer [12], and mechanical difficulties with the implant requiring revision [14].

**Table 5:** Summary of intraoperative and postoperative complications following primary and salvage distal femoral endoprosthetic replacement for traumatic distal femoral fractures.

Complication	Primary DFR(n = 230) (n, %)	Salvage DFR(n = 51) (n, %)	*P* value^a^
Intraoperative			
Cardiac arrest	1 (0.4)	0 (0)	1
Bone cement implantation syndrome	0 (0)	2 (3.9)	.032
Postoperative			
Superficial infection	6 (2.6)	5 (9.8)	.046
Deep infection	7 (3.0)	5 (9.8)	.075
Wound breakdown	0 (0)	0 (0)	n/a
Venousthromboembolism	3 (1.3)	1 (1.9)	.553
Dislocation	0 (0)	0 (0)	1
Extensor mechanismdisruption	1 (0.4)	0 (0)	1
Aseptic loosening	6 (2.6)	2 (3.9)	.639
Periprosthetic fracture	8 (3.5)	1 (1.9)	1
Stiffness requiringMUA	0 (0)	0 (0)	n/a
Critical care requirement	2 (0.9)	3 (5.9)	.043
Other	2 (0.9)	3 (5.9)	.043
Overall complication	36 (15.7)	22 (43.1)	

MUA, manipulation under anesthesia.

a Chi-square test, Fisher's exact test when n < 5.

Superficial infections occurred at a significantly increased rate in the pDFR cohort, while bone cement implantation syndrome, critical care requirements, and other complications were significantly more frequent in the sDFR cohort. Indications for critical care requirement included hypotension, respiratory failure, bone cement implantation syndrome, and hyponatremia [7,12].

**Figure 3:**
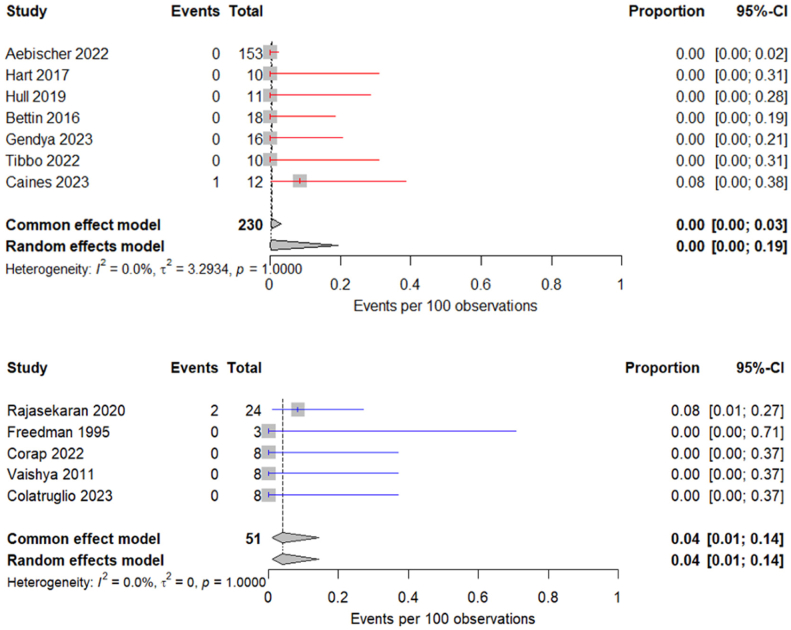
Forest plot illustrating the weighted intraoperative complication rate for studies evaluating primary (red) and salvage (blue) DFR.

**Figure 4:**
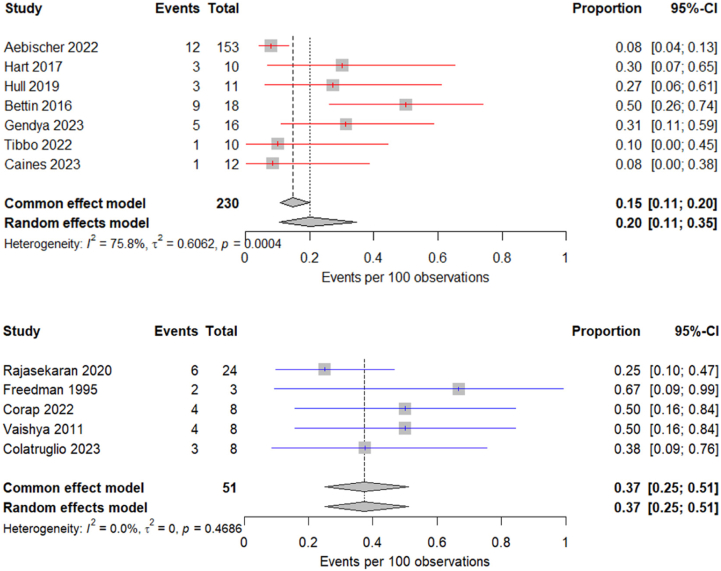
Forest plot illustrating the weighted postoperative complication rate for studies evaluating primary (red) and salvage (blue) DFR.

The pooled intraoperative complication rate for the pDFR cohort was 0% (95% CI: 0.0 to 3.0) and for the sDFR cohort was 4.0% (95% CI: 1.0 to 14.0) (Fig. 3). The pooled postoperative complication rate for the pDFR cohort was 20.0% (95% CI: 11.0 to 35.0) and for the sDFR cohort was 37.0% (95% CI: 25.0 to 51.0) (Fig. 4).

### Functional outcomes

**Figure 5:**
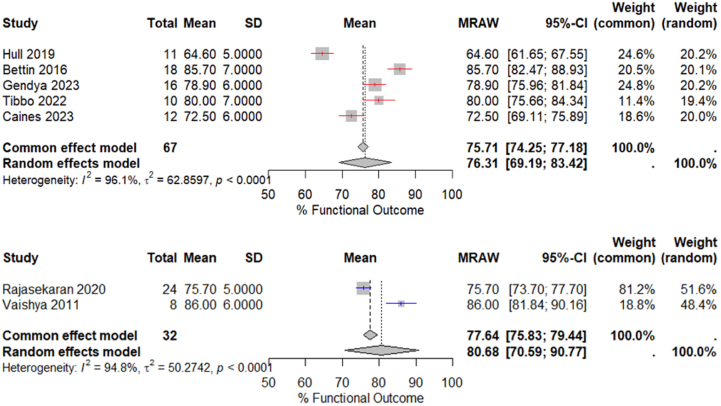
Forest plot illustrating the weighted means for functional outcome in studies evaluating primary (red) DFR (mean follow-up of 31.4 months) and salvage (blue) DFR (mean follow-up of 41.4 months).

The overall functional outcome scores were higher in the sDFR cohort. A weighted comparison of the pooled functional outcome scores between the groups found no statistically significant difference (76.3 vs 80.7%; *P* = .09; 95% CI: – 0.7 to 9.5) (Fig. 5). It is important to note that functional scores were not assessed at equivalent time points, and follow-up duration was significantly longer in the sDFR cohort.

## Discussion

The most important finding of this study is that sDFR following failed fixation of DFFs was associated with nearly double the risk of reoperation and postoperative complications compared to pDFR, despite being in a younger and less comorbid cohort. We provide the first review that presents a pooled analysis of the literature evaluating the outcomes of pDFR and sDFR following native DFFs. Previous studies comparing primary ORIF and DFR for native DFFs have demonstrated high nonunion rates of up to 20-24% following fixation and a trend toward lower revision and reoperation rates with DFR [5,6]. This is particularly the case in elderly patients, where lower preinjury activity levels, poor bone stock, and higher comorbidity profiles contribute to poorer outcomes after fixation [7]. This study builds upon those findings by highlighting that sDFR following ORIF comes with a much higher risk profile compared with a pDFR. The significantly higher reoperation and revision rates observed in the sDFR cohort highlight the technical challenges of salvage surgery and the potential advantages of primary arthroplasty in selected patients at high risk of fixation failure. This pattern is consistent with findings in other periarticular fractures, such as tibial plateau fractures, where primary total knee arthroplasty has also been associated with fewer complications compared to secondary total knee arthroplasty following failed fixation [19]. Surgeons should carefully consider this increased risk when managing complex DFFs, particularly when choosing between primary fixation vs pDFR.

Managing nonunion of the distal femur is complex due to fibrosis, knee stiffness and difficulty in surgical exposure [11,12]. While pDFR is a viable alternative, the restricted salvage options if revision surgery is required, and the potentially devastating sequelae of infection in a DFR must be highlighted. Cost is often cited as a perceived disadvantage of DFR compared with ORIF, particularly due to the higher implant cost. Gwam et al. [20] reported significantly higher costs for DFR ($46,323 vs $7515), though their analysis was limited by the inability to disaggregate individual cost components, making it unclear which factors contributed most to these differences. Similarly, Brodke et al. [21] conducted a cost-effectiveness analysis which found fixation to be more cost-effective ($25,556 vs $65,536), but this model was informed by small, underpowered series that may not accurately reflect true cost-effectiveness. Caines et al. [9] reported significantly higher implant ($11,403 vs $2066) and overall cost ($61,259 vs $44,490) but demonstrated no significant difference in overall costs once the index procedure, length of stay, and revision surgeries were included. On face value, DFR appears more expensive, but its overall cost-effectiveness remains uncertain. Current literature is limited to small case series with heterogeneous reporting, and few studies account for broader healthcare utilization beyond implant cost, precluding meaningful economic conclusions. Larger observational studies and well-designed clinical trials are needed to clarify the true economic impact of DFR vs ORIF, as both procedures may offer benefits not adequately captured in the existing literature.

Early mobilization is cited by many as a key advantage of DFR. Though immediate weight-bearing is safe following lateral locking plate fixation of the distal femur [22], weight-bearing restrictions are often imposed by surgeons [23] especially for fractures with articular comminution. Immediate unrestricted weight-bearing, permitted after pDFR has been shown to significantly reduce morbidity and mortality in elderly patients [24].

In line with common practice, we observed that pDFR was more commonly performed in older patients. Concerns regarding the lifetime risk of revision of DFR for mechanical failure, especially aseptic loosening, typically limit the use of endoprostheses to older patients with limited life expectancies, except where no other reconstructive option is available. Older adults are more likely to have poor bone stock and high levels of comminution that may render fractures unreconstructable and more likely, therefore to be treated with DFR in preference to fixation. Increased comorbidities and reduced rehabilitation potential may also lead surgeons toward pDFR to mitigate the risk of nonunion, postoperative complications associated with weight-bearing restrictions, and subsequent revision surgery.

Despite the exclusion of 2 pDFR studies reporting mortality at 4.7 years and prior to 2 years, respectively [6,7], the 1-year mortality rate remained significantly higher in the pDFR cohort. This is likely reflective of the older age and greater comorbidity of these patients, and surgeons are more likely to choose DFR in frail elderly patients with unreconstructable fractures, introducing an inherent selection bias. Most included studies did not detail causes of death, but where reported, deaths appeared unrelated to the fracture or intervention [7]. The mortality difference is best interpreted as a reflection of patient frailty rather than the procedure itself, and a direct comparison with the younger, healthier sDFR cohort should be made with caution.

Despite being younger and less comorbid, patients in the sDFR cohort experienced significantly higher complication rates. sDFR is surgically more complex, requiring implant removal, debridement and management of soft tissues and scarring, all of which prolong operative time and increase the risk of hemodynamic instability. Four studies reported increased intraoperative blood loss in the sDFR cohort [6,7,11,12], consistent with previous literature indicating that salvage procedures are associated with increased transfusion requirements and greater morbidity [25].

The length of stay was longer in the pDFR cohort, although this difference was not statistically significant. While one might expect pDFR patients to mobilize and discharge earlier, older and more comorbid patients often experience delayed recovery and more complex discharge planning which can prolong hospitalization, which may account for this observation [26].

Functional outcomes were marginally lower in the pDFR cohort, although this difference was not statistically significant. Several factors may explain this observation. First, functional assessments were not performed at equivalent time points, with sDFR patients having a significantly longer follow-up duration, providing more time for rehabilitation and functional recovery. Second, survivorship bias is also relevant, as functional scores are typically reported only for patients who survived and could reflect a self-selecting group who are more physiologically resilient and therefore confer greater functional outcomes which may not be representative of the cohort as a whole. Given the higher mortality in the pDFR cohort, poorer outcomes may be underrepresented. Finally, patients undergoing pDFR were typically older and more comorbid, whereas those requiring sDFR were often younger and healthier at the time of their index fracture. This demographic difference potentially contributed to the slightly higher functional scores observed in the sDFR cohort, despite their higher complication and reoperation rates.

The authors acknowledge that the pDFR and sDFR cohorts differ in baseline demographics: pDFR is typically performed in older, more comorbid, and predominantly female patients, while sDFR is more common in younger, healthier individuals, with more gender balance. This comparison does not reflect a direct clinical decision since clinicians do not typically choose between pDFR and sDFR at the time of injury. Instead, the aim was to explore the differences in outcomes between the 2 approaches to inform the consequences of initial management strategy.

This study has limitations. These cases are relatively unusual, and the case series were largely retrospective with small sample sizes by arthroplasty standards, particularly in the sDFR cohort, and therefore susceptible to type II error. This limits interpretation and necessitates caution when drawing firm conclusions regarding secondary outcomes. The authors recommend that further research studies incorporating multicenter cohorts or registry-based data should be employed. Both reporting and publishing bias are present. Centers that have attempted pDFR for DFFs with poor outcomes are unlikely to have published them. There was considerable variability in patient selection, surgical technique, implant choice and rehabilitation protocols, as well as in how outcomes such as functional scores, intraoperative blood loss, and transfusion requirements were reported, which limited the ability to make direct comparisons. Data on the interval between ORIF and sDFR are limited, with only 2 of the five studies reporting this information. All included studies stated that patients undergoing DFR were permitted immediate or postoperative full weight-bearing. However, none explicitly detailed the exact interval to mobilization. These gaps highlight the need to establish minimum reporting standards in future studies evaluating both pDFR and sDFR to improve comparability across the literature. Follow-up durations were not standardized across studies, making it difficult to draw meaningful conclusions in this regard. Most studies included in our review were evaluated to be of low to moderate quality. No studies presented prefracture patient-reported outcome measures, limiting the ability to quantify the degree of return to preinjury functional status. Furthermore, health-related quality of life outcomes was not reported by any studies, representing an important gap in the literature that should be addressed in future research.

## Conclusions

This systematic review comparing the available evidence on primary vs salvage DFR for DFFs highlights the increased risks associated with sDFR following failed ORIF compared with pDFR, with nearly double the rate of reoperation and postoperative complications compared with pDFR. Identifying risk factors for fixation failure, such as patient factors, fracture patterns, and concerns regarding fixation quality, is critical. In selected patients where these risks are considerable, surgeons could consider pDFR as a suitable initial management to avoid the morbidity associated with salvage procedures. Although such decisions are nuanced, a careful, patient-centered approach, balancing the benefits of native joint preservation against the risks of fixation failure, is crucial to optimizing outcomes. Future research should aim to refine patient selection criteria and clarify optimal indications in the management of native DFFs.

## Conflicts of interest

C.E.H. Scott is a paid consultant for Stryker, Smith and Nephew, and Osstec; owns stock or stock options in Osstec; is PI on an institutional research grant from Stryker; is an Editor-in-Chief of *Bone and Joint Research*; and a member of the editorial board for *Bone and Joint Journal*. N.D. Clement is a board member of the *Bone and Joint Journal* and *Bone and Joint Research*; all other authors declare no potential conflicts of interest.

For full disclosure statements refer to https://doi.org/10.1016/j.artd.2025.101939.

## CRediT authorship contribution statement

**Lucas Ho:** Writing – review & editing, Writing – original draft, Visualization, Validation, Software, Resources, Project administration, Methodology, Investigation, Formal analysis, Data curation, Conceptualization. **Navnit S. Makaram:** Writing – review & editing, Writing – original draft, Visualization, Validation, Supervision, Methodology, Investigation, Data curation, Conceptualization. **Catherine James:** Writing – review & editing, Visualization, Supervision. **Chryssa Neo:** Writing – review & editing, Visualization, Supervision. **Nick D. Clement:** Writing – review & editing, Writing – original draft, Visualization, Validation, Supervision, Methodology, Investigation. **Chloe E.H. Scott:** Writing – review & editing, Writing – original draft, Visualization, Validation, Supervision, Methodology, Investigation, Conceptualization.

## Appendix A . Supplementary data

Conflict of Interest Statement for Scott

Conflict of Interest Statement for James

Conflict of Interest Statement for Neo

Conflict of Interest Statement for Ho

Conflict of Interest Statement for Clement

Conflict of Interest Statement for Makaram
