# Supplementary material for: Primary Versus Salvage Distal Femoral Endoprosthetic Replacement Following Native Distal Femur Fracture: A Systematic Review and Meta-Analysis
Source: Arthroplast Today. 2026 Jan 7;37:101939. doi: 10.1016/j.artd.2025.101939 (PMC12809088; doi:10.1016/j.artd.2025.101939)
Supplement: Conflict of Interest Statement for Scott [file mmc1.docx]

# INDIVIDUAL CONFLICT OF INTEREST STATEMENT

***American Association of Hip and Knee Surgeons***

(Adopted from the American Academy of Orthopaedic Surgeons disclosure statement)

The following form **must be filled out completely and submitted by each author (example, 6 authors, 6 forms).**

**All items require a response. If there is no relevant disclosure for a given item, enter "*None*.”**

**Primary versus salvage distal femur endoprosthetic replacement following native distal femur fracture in the elderly: a systematic review and meta-analysis**

**Manuscript Title**

1. Royalties from a company or supplier (The following conflicts were disclosed) None

2. Speakers bureau/paid presentations for a company or supplier (The following conflicts were disclosed)

None

3A. Paid employee for a company or supplier (The following conflicts were disclosed) None

3B. Paid consultant for a company or supplier (The following conflicts were disclosed)

Stryker, Smith and Nephew, Osstec

3C. Unpaid consultants for a company or supplier (The following conflicts were disclosed) None

4. Stock or stock options in a company or supplier (The following conflicts were disclosed)

Osstec

5. Research support from a company or supplier as a Principal Investigator (The following conflicts were disclosed)

PI on an institutional research grant from Stryker

6. Other financial or material support from a company or supplier (The following conflicts were disclosed)

None

7. Royalties, financial or material support from publishers (The following conflicts were disclosed)

None

8. Medical/Orthopaedic publications editorial/governing board (The following conflicts were disclosed)

Editor-in-Chief Bone and Joint Research; Editorial Board Bone and Joint Journal

9. Board member/committee appointments for a society (The following conflicts were disclosed)

None

**Each author must sign AND print or type his/her name, date and submit a separate form**

In addition, one BLINDED Conflict of Interest form (no author names used) should be submitted per manuscript with all author disclosures.

Chloe Scott
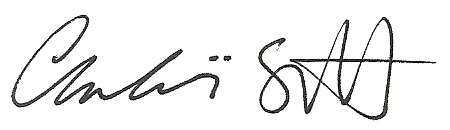
 17/06/2025

Author Name (Print or Type) Author Signature Date
